# Supplementary material for: The combined action of the intracellular regions regulates FGFR2 kinase activity
Source: Commun Biol. 2023 Jul 14;6:728. doi: 10.1038/s42003-023-05112-6 (PMC10349056; doi:10.1038/s42003-023-05112-6)
Supplement: Supplementary file 2 — Supplementary Information [file 42003_2023_5112_MOESM2_ESM.pdf]

The combined action of the intracellular regions regulates FGFR2 kinase activity

Chi-Chuan Lin, Lukasz Wieteska, Guillaume Poncet-Montange, Kin Man Suen, Stefan T. Arold, Zamal Ahmed, John E. Ladbury.

Supplementary information includes six Figures and seven Tables.

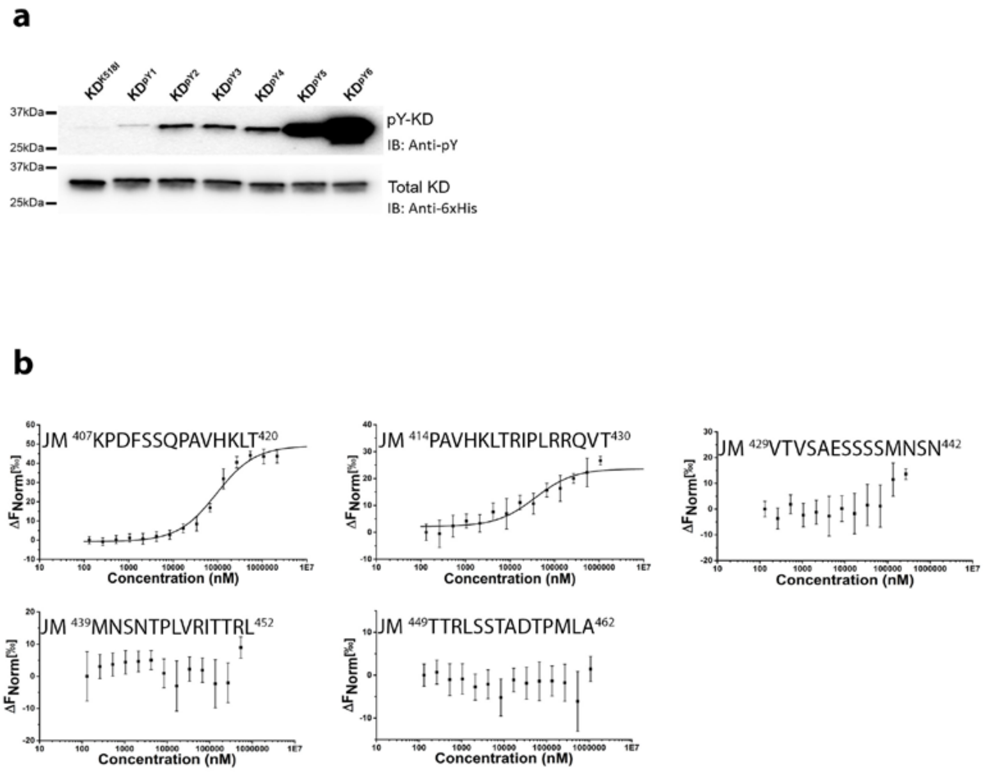
a
KD K518I
KD pY1
KD pY2
KD pY3
KD pY4
KD pY5
KD pY6
37kDa
25kDa
pY-KD
IB: Anti-pY
Total KD
IB: Anti-6xHis
b
JM 407KPDFSSQPAVHKLT420
JM 414PAVHKLTRIPLRRQVT430
JM 429VTVSAESSSSMNSN442
JM 439MNSNTPLVRITTRL452
JM 449TTRLSSTADTPMLA462
ΔFNorm[‰]
Concentration (nM)

Supplementary Fig. 1

The interaction of JM with KD.

a. The phosphorylation states of KD^{pY1} to KD^{pY6} were confirmed using a phosphotyrosine pY99 antibody. An anti-6xHis tag antibody was used to probe for total proteins as the loading control.

b. Five short JM peptides were synthesised (residues 407-420, 414-430, 429-442, 439-452, and 449-462) and used to identify the binding region for KD^{pY1}. The MST measurement results indicate that residue 407-420 provides the best binding ability for KD^{pY1}. The error bars are presented as the standard deviation.

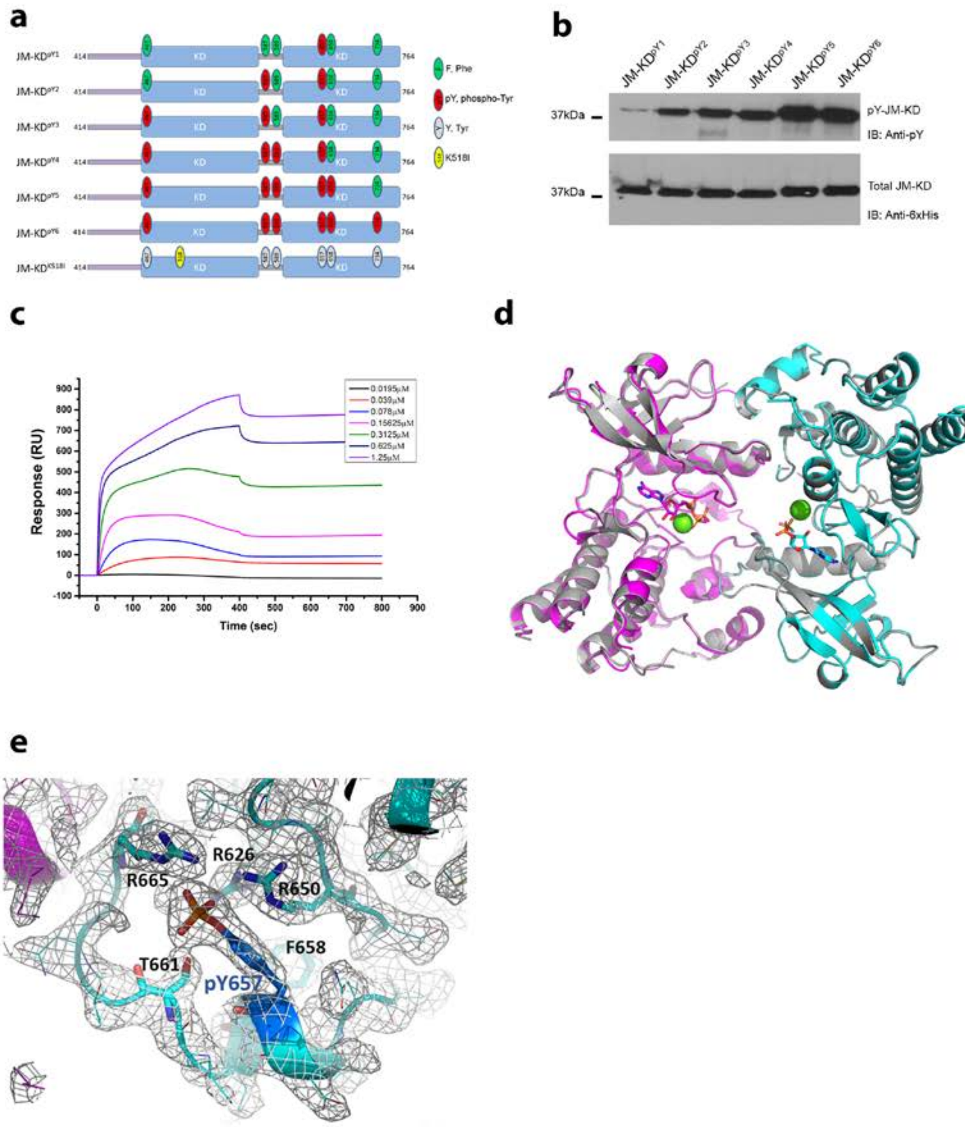
a
JM-KDpY1
JM-KDpY2
JM-KDpY3
JM-KDpY4
JM-KDpY5
JM-KDpY6
JM-KDK518I
414
KD
764
F, Phe
pY, phospho-Tyr
Y, Tyr
K518I
b
JM-KDpY1
JM-KDpY2
JM-KDpY3
JM-KDpY4
JM-KDpY5
JM-KDpY6
37kDa
pY-JM-KD
IB: Anti-pY
37kDa
Total JM-KD
IB: Anti-6xHis
c
Response (RU)
Time (sec)
0.0195μM
0.039μM
0.078μM
0.15625μM
0.3125μM
0.625μM
1.25μM
d
e
R626
R665
R650
F658
T661
pY657

Supplementary Fig. 2

Dimerization of mono-phosphorylated kinase.

a. Schematic of JM-KD with progressively phosphorylation KD (FGFR2$_{414\text{-}764}$). Six tyrosine residues on KD were mutated to mimic the sequential phosphorylation pattern of KD (JM-KD^{pY1} to JM-KD^{pY6}).

b. Phosphorylation states of JM-KD^{pY1} to JM-KD^{pY6} were examined using a phosphotyrosine pY99 antibody. An anti-6xHis tag antibody was used to probe for total proteins as the loading control.

c. Dimerization of JM-KD^{pY1} was examined using surface plasmon resonance (SPR). JM-KD^{pY1} was immobilised on a CM4 chip by amine coupling, a serial dilution of JM-KD^{pY1} was injected for 400 seconds and washed with buffer for further 400 seconds. The binding affinity was calculated using steady-state fitting model.

d. X-ray crystal structural detail showing the asymmetric unit of KD^{pY1} containing four molecules. Phosphorylated chains A (magenta) and C (cyan) superimposed onto chains C and D (grey). ATP shown as stick model, and Mg^{2+} as green sphere.

e. A-loop (chain B) in its 2FoFc electron density derived from X-ray crystal structure of asymmetric unit of KD^{pY1}.

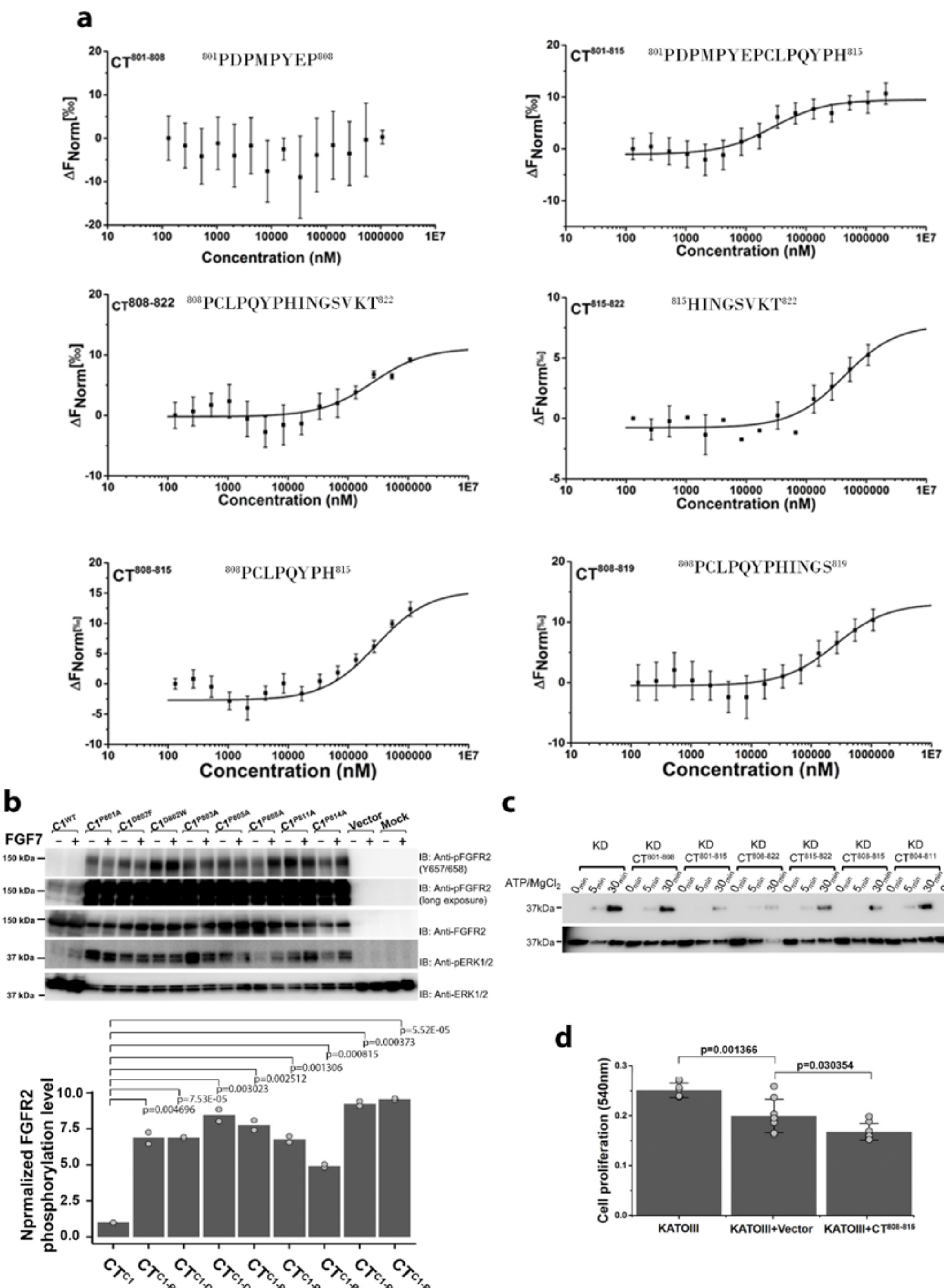
a
CT801-808 801PDPMPYEP808
CT801-815 801PDPMPYEPCLPQYPH815
CT808-822 808PCLPQYPHINGSVKT822
CT815-822 815HINGSVKT822
CT808-815 808PCLPQYPH815
CT808-819 808PCLPQYPHINGS819
ΔFNorm[‰]
Concentration (nM)
b
FGF7
C1WT
C1P801A
C1D802F
C1D802W
C1P803A
C1P805A
C1P808A
C1P811A
C1P814A
Vector
Mock
150 kDa
37 kDa
IB: Anti-pFGFR2 (Y657/658)
IB: Anti-pFGFR2 (long exposure)
IB: Anti-FGFR2
IB: Anti-pERK1/2
IB: Anti-ERK1/2
p=5.52E-05
p=0.000373
p=0.000815
p=0.001306
p=0.002512
p=0.003023
p=7.53E-05
p=0.004696
Nprmalized FGFR2 phosphorylation level
CTC1
CTC1-P801A
CTC1-D802F
CTC1-D802W
CTC1-P803A
CTC1-P805A
CTC1-P808A
CTC1-P811A
CTC1-P815A
c
KD
KD CT801-808
KD CT801-815
KD CT808-822
KD CT815-822
KD CT808-815
KD CT804-811
KD CT808-819
ATP/MgCl2
0min
5min
30min
37kDa
IB: Anti-pY (pY-KD)
IB: Anti-His (total KD)
d
p=0.001366
p=0.030354
Cell proliferation (540nm)
KATOIII
KATOIII+Vector
KATOIII+CT808-815

Supplementary Fig. 3

CT^{C1} fragments bind to KD^{pY1}.

a. Binding of small CT peptides (801-808, 801-815, 808-822, 815-822, 808-815, and 808-819) to KD^{pY1} using MST. Sequences are shown of peptide shown. The error bars are presented as the standard deviation.

b. The presence of the intact proline-rich motif in $FGFR2^{C1}$ inhibits both FGFR2 and downstream ERK1/2 activities. $FGFR2^{C1}$ variants with individual P/A, D/F, and D/W mutants as indicated were transfected into HEK293T cells. Cells were starved or stimulated with 10ng/ml FGF7 for 15 minutes. Cell lysates were blotted with indicated antibodies to examine the importance of the proline-rich sequence on CT. Densitometric graph represents 2 independent experiments.

c. Dephosphorylated KD was incubated with seven CT-derived short peptides including fragments of the proline-rich sequences (residues 801-808; 801-815; 808-822; 815-822; 808-815; 804-811 and 808-819) to test their ability to regulate kinase activity. See Methods for phosphorylation and quenching procedures.

d. MTT assay of expression peptide 080-815 in KATOIII cell line. Untreated cells and cells with the empty vector expression were used as the controls. Densitometric graph represents results from 8 samples. The error bars are presented as the standard deviation.

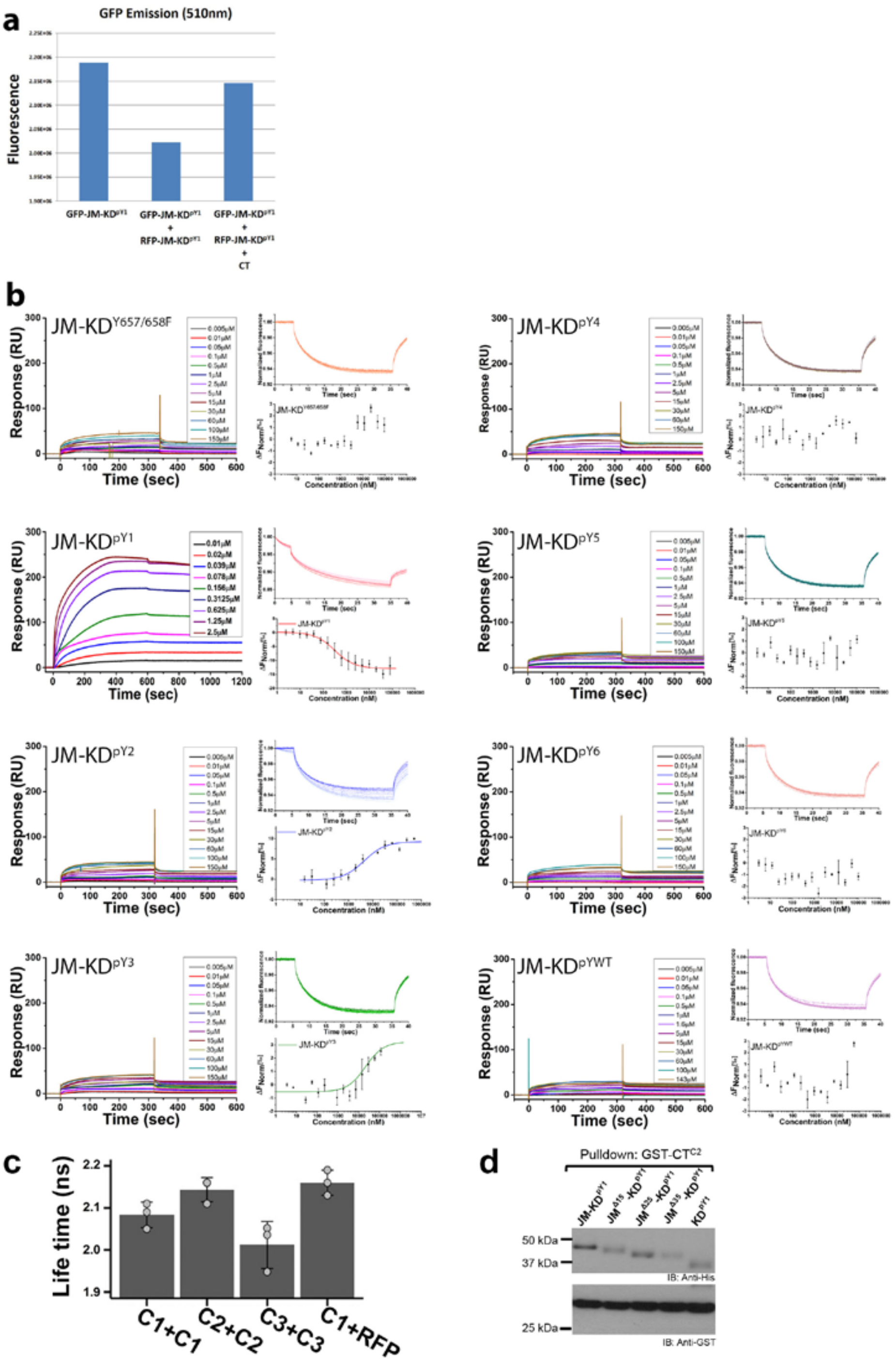
a
GFP Emission (510nm)
Fluorescence
GFP-JM-KDpY1
GFP-JM-KDpY1 + RFP-JM-KDpY1
GFP-JM-KDpY1 + RFP-JM-KDpY1 + CT
b
JM-KDY657/658F
JM-KDpY1
JM-KDpY2
JM-KDpY3
JM-KDpY4
JM-KDpY5
JM-KDpY6
JM-KDpYWT
Response (RU)
Time (sec)
Concentration (nM)
c
Life time (ns)
C1+C1
C2+C2
C3+C3
C1+RFP
d
Pulldown: GST-CTC2
50 kDa
37 kDa
25 kDa
IB: Anti-His
IB: Anti-GST

Supplementary Fig. 4

Phosphorylation states control KD and CT^{C1} interaction.

a. Steady-state FRET study using GFP and RFP tagged JM-KD^{pY1} demonstrates that the dimer formation of GFP and RFP tagged JM-KD^{pY1} as indicated by the decrease of FRET donor emission (510nm). In addition, the binding of CT to JM-KD^{pY1} results in the dissociation of the asymmetric JM-KD^{pY1} dimer as indicated by the recovery of FRET donor emission (510nm).

b. The phosphorylation levels of JM-KD tightly control the interaction with CT. For the SPR experiments, untagged CT (the last 58 residues of FGFR2) was immobilised on a CM4 chip via amine coupling. Kinases with different phosphorylation level (JM-KD^{pY1} to JM-KD^{pY6}, and JM-KD^{pYWT}, and JM-KD$^{Y657/658F}$) were injected followed by a buffer wash. The binding affinities were determined using stead-state fitting. For the MST measurements, untagged CT was labelled by Atto 488. Two-fold serial dilutions of kinase proteins as described above were used to mix with labelled CT (100nM) and the binding affinities were determined. Both SPR and MST experiments show that the mono-phosphorylated JM-KD is the strongest binding partner for the CT. These experiments provide direct evidence of KD-CT interaction at the basal state. The error bars are presented as the standard deviation. Both MST and SPR graphs for JM-KD^{pY1} are also presented in Fig. 3c and Fig.4b

c. Statistics bar graph analysis of FGFR2 isomerization using FLIM. Representative of 3 independent experiments. The error bars are presented as the standard deviation.

d. GST-CT^{C2} was used to pull down five mono-phosphorylated constructs of JM-KD^{pY1} as described in Fig. 6D. The presence of the intact JM also enhances the interaction with the CT from C2 isoform.

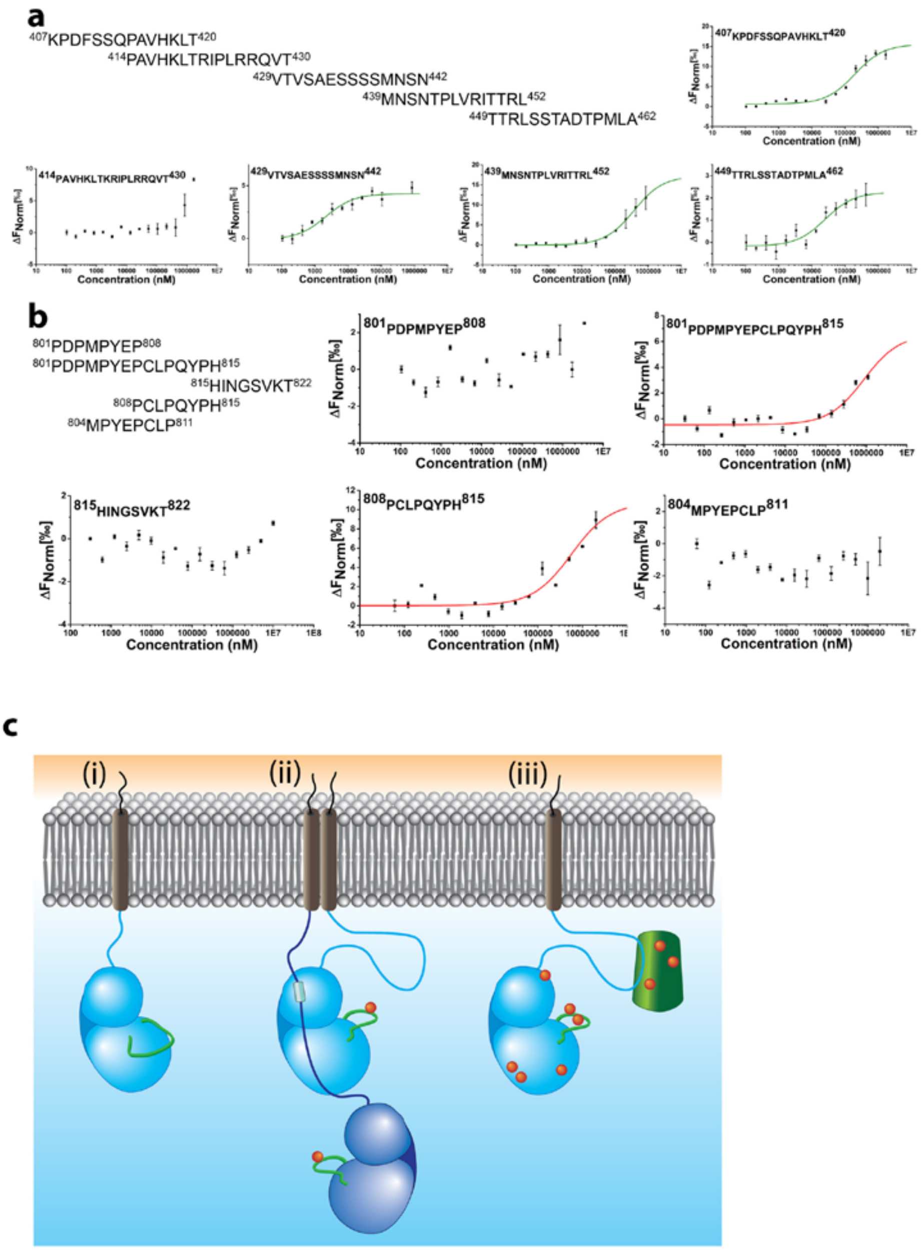
a
407KPDFSSQPAVHKLT420
414PAVHKLTRIPLRRQVT430
429VTVSAESSSSMNSN442
439MNSNTPLVRITTRL452
449TTRLSSTADTPMLA462
407KPDFSSQPAVHKLT420
414PAVHKLTKRIPLRRQVT430
429VTVSAESSSSMNSN442
439MNSNTPLVRITTRL452
449TTRLSSTADTPMLA462
ΔFNorm[‰]
Concentration (nM)
b
801PDPMPYEP808
801PDPMPYEPCLPQYPH815
815HINGSVKT822
808PCLPQYPH815
804MPYEPCLP811
801PDPMPYEP808
801PDPMPYEPCLPQYPH815
815HINGSVKT822
808PCLPQYPH815
804MPYEPCLP811
ΔFNorm[‰]
Concentration (nM)
c
(i)
(ii)
(iii)

Supplementary Fig. 5

CT-JM interactions

a. Small JM peptides (residues 407-420, 414-430, 429-442, 439-452, and 449-462) were used for the MST measurements with CT. CT was labelled with Atto 488 and 2-fold serial dilutions of small JM peptides were mixed with 100 nM labelled CT. The error bars are presented as the standard deviation.

b. Small CT peptides (residues 801-808, 801-815, 815-822, 808-815, and 804-811) were used for the MST measurements with JM. JM was labelled with Atto488 and 2-fold serial dilutions of small CT peptides were mixed with 100 nM labelled JM. The error bars are presented as the standard error of the mean.

c. Schematic representation of activation of $FGFR2^{C3}$ K*sam* mutant. i: In the absence of stimulation the unphosphorylated $FGFR2^{C3}$ (light blue, JM light blue line) can exist as a monomer freely diffusing through the plasma membrane. ii: Under normal expression levels in non-stimulated cells FGFR2 will self-associate through random collision. Such collision between $FGFR2^{C3}$ molecules in the absence of negative control of CT results in formation A-loop phosphorylation (red spot on green line) and interaction of JM latch and asymmetric dimerization to form active enzyme. (Enzyme-like receptor: dark blue, substrate-like receptor: light blue). iii) Active asymmetric dimerization leads to trans-autophosphorylation (red spots) of $FGFR2^{C3}$ which is unrestrained by CT. The conformational change associated with phosphorylation of the KD enables recruitment and phosphorylation of FRS2 (green shape) which provides the required site for subsequent recruitment of downstream signalling effector proteins to initiate signal transduction.

1a

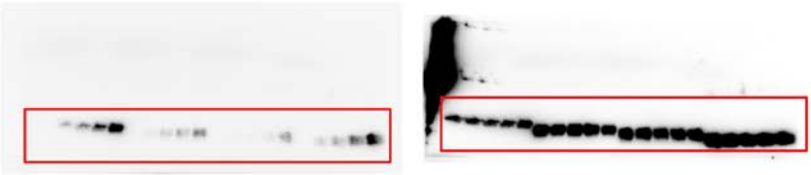

1b

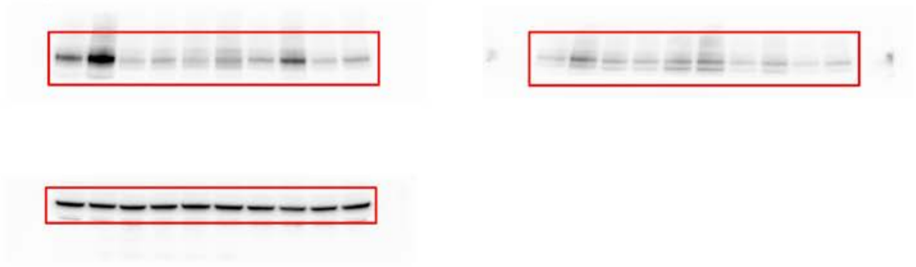

1c

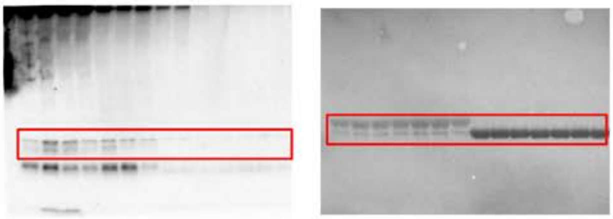

2d

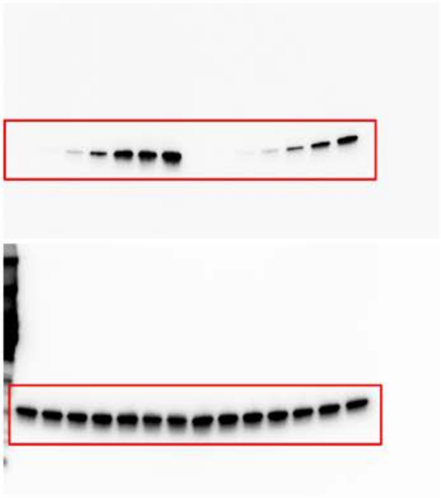

2e

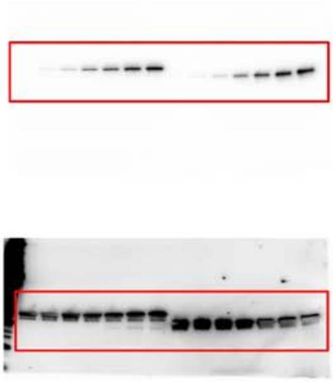

3a

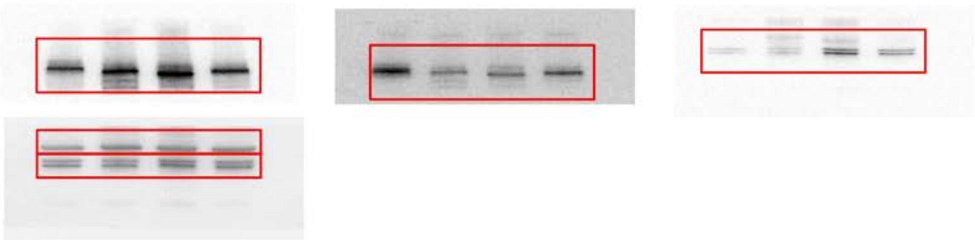

3b

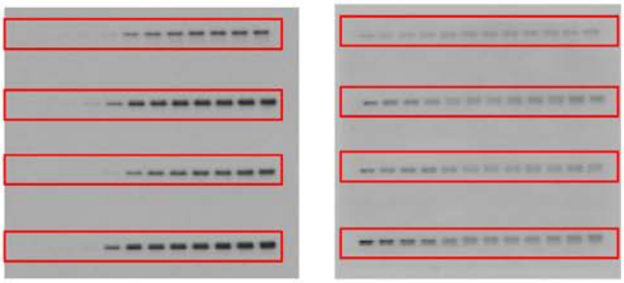

3e

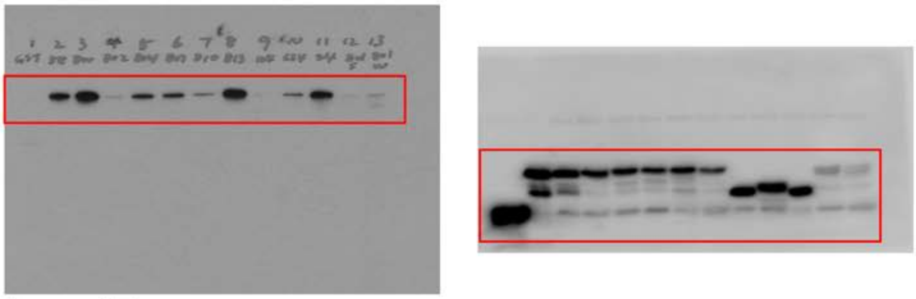

4a and 5e

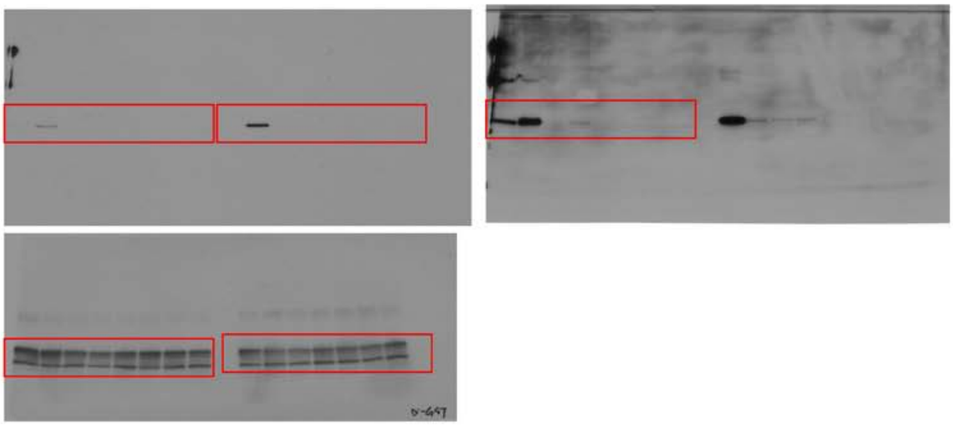

4d

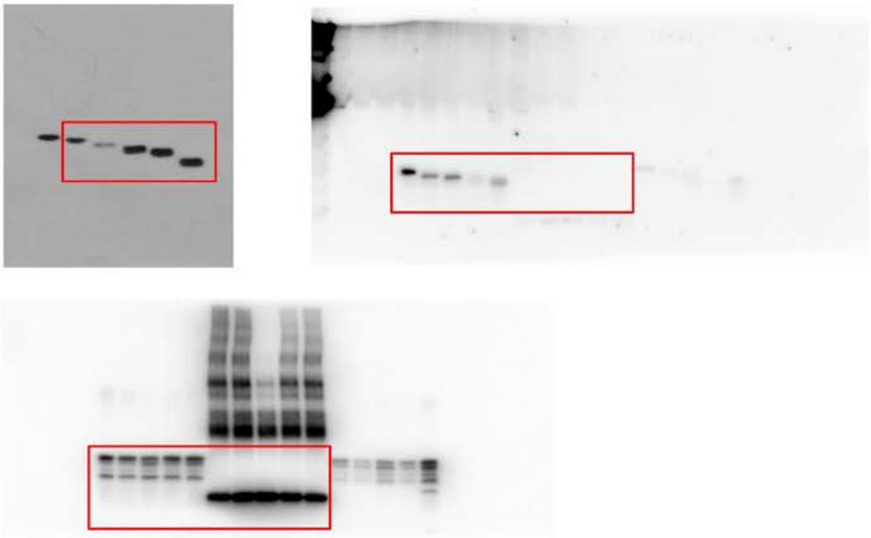

4e

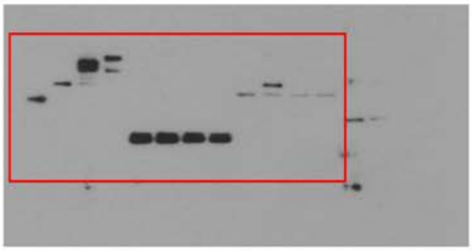

4f

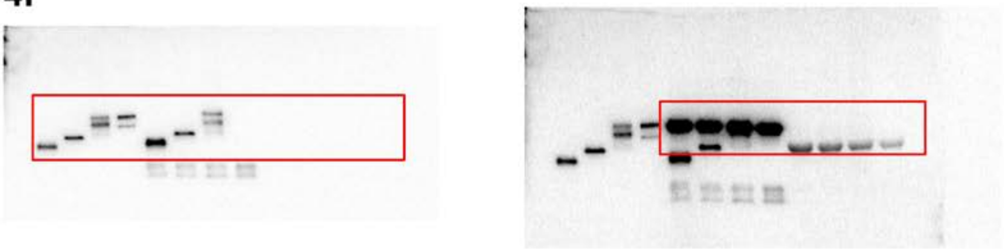

5a

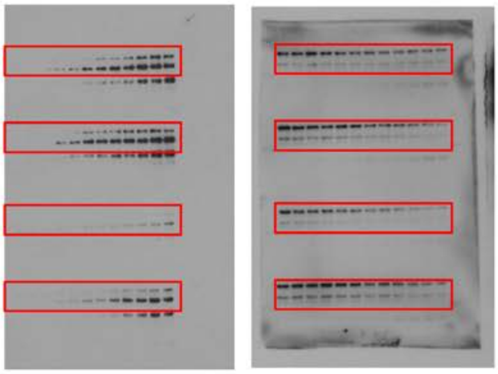

5c

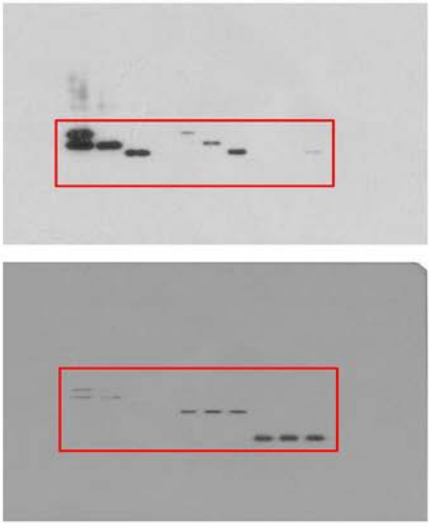

5e

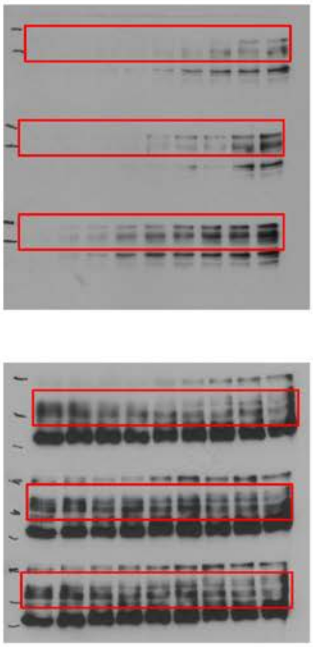

s1a

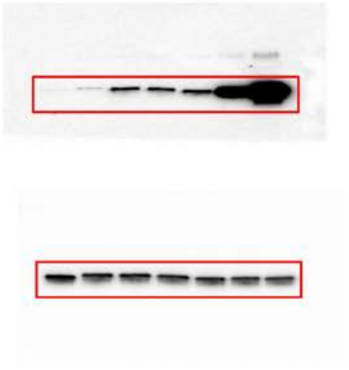

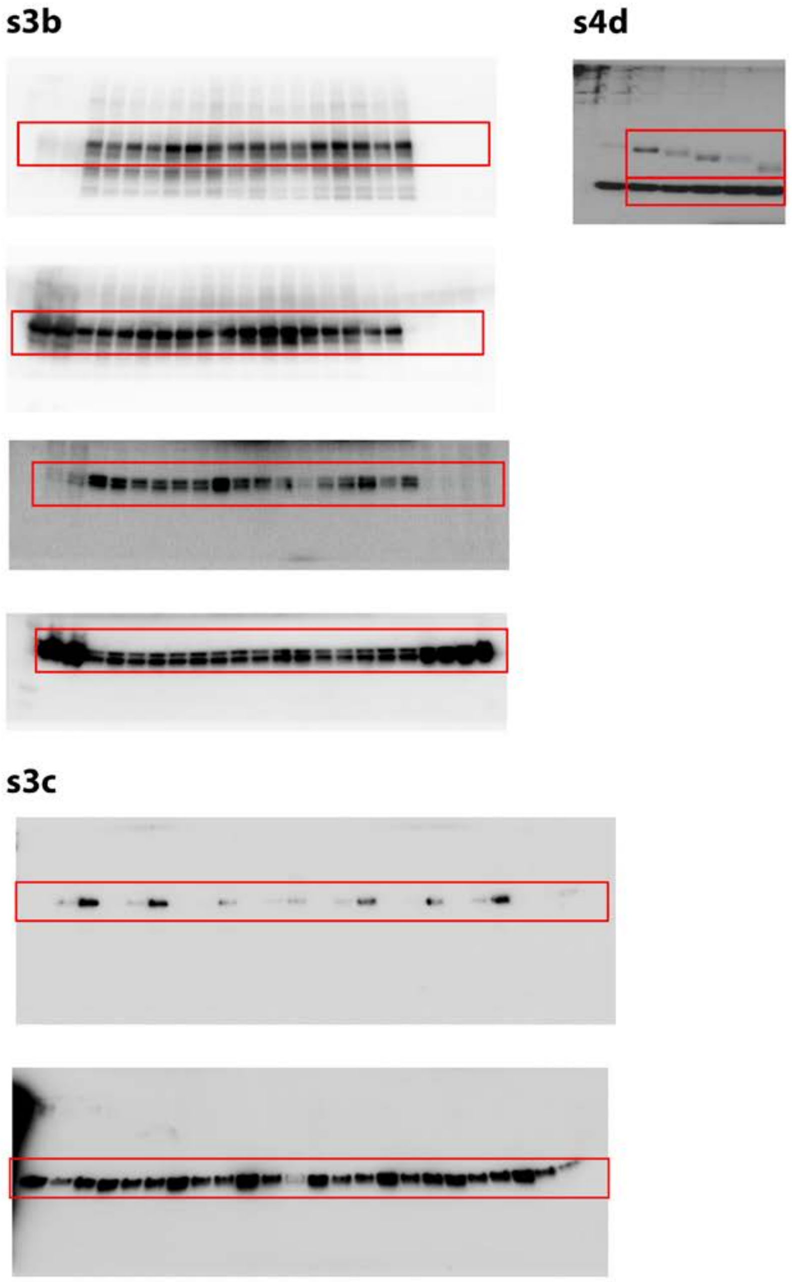

Supplementary Fig. 6

Original images for the immunoblotting experiment shown in this study

Supplementary Table 1 Binding affinities of KD with progressively increased phosphorylation states to JM measured by microscale thermophoresis (MST). Related to Fig. 1c.

FGFR2 Kinase proteins	Affinity to JM
KD^{K518I}	13.4±0.994 µM
KD^{pY1}	2.51±0.203 µM
KD^{pY2}	6.98±0.877µM
KD^{pY4}	10.2±1.33 µM
KD^{pY5}	22.5±1.8 µM
KD^{p6}	20.5±2.04 µM

Supplementary Table 2 Binding affinities of JM and its short peptides to KD^{pY1} measured by microscale thermophoresis (MST). Related to Supplementary Fig. 1c.

JM sequences	Affinity to KD^{pY1}
^{407}Juxtamembrane region^{462}	2.51±0.203 µM, Table S1
^{407}KPDFSSQPAVHKLT^{420}	105±7.37 µM
^{414}PAVHKLTKRIPLRRQVT^{430}	36.8±6.23 µM
^{429}VTVSAESSSSMNSN^{442}	Very weak
^{439}MNSNTPLVRITTRL^{452}	No binding
^{449}TTRLSSTADTPMLA^{462}	No binding

Supplementary Table 3 Data collection and refinement statistics (molecular replacement). Related to Supplementary Fig. 2d, e.

	FGFR2IIIb (414-822), PTR657 (PDB6V6Q)
**Data collection**	
Space group	$P\ 2_1\ 2_1\ 2_1$
Cell dimensions	
a, *b*, *c* (Å)	64.0, 86.5, 254.2
α, β, γ (°)	90 ,90 ,90
Resolution (Å)	81.92-2.46 (2.55-2.46) *
R_{merge}	0.098 (1.088)
$I / \sigma I$	12.7 (1.8)
Completeness (%)	96.6 (80.3)
Redundancy	6.7 (3.6)
**Refinement**	
Resolution (Å)	81.92-2.46
No. reflections	50128 (3761)
R_{work} / R_{free}	21.49/23.88
No. atoms	
Protein	9,018
Ligand/ion	124
Water	125
B-factors	
Protein	72.16
Ligand/ion	83.82
Water	49.06
R.m.s. deviations	
Bond lengths (Å)	0.007
Bond angles (°)	1.39

Supplementary Table 4 Binding affinities of short C-terminal tail peptides derived from CT^{C1} to KD^{pY1} measured by microscale thermophoresis (MST). Related to Supplementary Fig. 3a.

**CT sequences**	**Affinity to KD^{pY1}**
^{801}PDPMPYEP^{808}	No binding
^{801}PDPMPYEPCLPQYPH^{815}	25.8±5.4 µM
^{808}PCLPQYPHINGSVKT^{822}	257±39.2 µM
^{815}HINGSVKT^{822}	426±42.5 µM
^{808}PCLPQYPH^{815}	291±21.4 µM
^{808}PCLPQYPHINGS^{819}	255±37.9 µM

Supplementary Table 5 Binding affinities of KD with progressively increasing phosphorylation states to CT^{C1} measured by surface plasmon resonance (SPR). Related to Fig. 4b and Supplementary Fig. 4b.

FGFR2 Kinase proteins	Affinity to CT^{C1}	SE(K_d)
$JM\text{-}KD^{Y657/658F}$	5.68 µM	1.9 µM
$JM\text{-}KD^{pY1}$	0.16 µM	0.02 µM
$JM\text{-}KD^{pY2}$	4.08 µM	0.50 µM
$JM\text{-}KD^{pY3}$	3.27 µM	0.80 µM
$JM\text{-}KD^{pY4}$	7.87 µM	1.70 µM
$JM\text{-}KD^{pY5}$	1.60 µM	0.20 µM
$JM\text{-}KD^{pY6}$	4.63 µM	0.91 µM
$JM\text{-}KD^{pWT}$	2.31 µM	0.48 µM

Supplementary Table 6 Binding affinities of short JM peptides derived from JM to CT^{C1} measured by microscale thermophoresis (MST). Related to Supplementary Fig. 5a.

JM sequences	Affinity to CT^{C1}
^{407}Juxtamembrane region^{462}	20.2±2.92 µM
^{407}KPDFSSQPAVHKLT^{420}	200±13.7 µM
^{414}PAVHKLTKRIPLRRQVT^{430}	No binding
^{429}VTVSAESSSSMNSN^{442}	3.23±0.326 µM
^{439}MNSNTPLVRITTRL^{452}	372±30.3 µM
^{449}TTRLSSTADTPMLA^{462}	25.4±3.26 µM

Supplementary Table 7 Binding affinities of short C-terminal tail peptides derived from CT^{C1} to JM measured by microscale thermophoresis (MST). Related to Supplementary Fig. 5b.

**CT^{C1} sequences**	**Affinity to JM**
^{801}PDPMPYEP^{808}	No binding
^{801}PDPMPYEPCLPQYPH^{815}	63.6±6.5 µM
^{815}HINGSVKT^{822}	No binding
^{808}PCLPQYPH^{815}	56.6±5.5 µM
^{804}MPYEPCL^{P811}	No binding
