# Supplementary material for: The combined action of the intracellular regions regulates FGFR2 kinase activity
Source: Commun Biol. 2023 Jul 14;6:728. doi: 10.1038/s42003-023-05112-6 (PMC10349056; doi:10.1038/s42003-023-05112-6)
Supplement: Supplementary file 3 — Description of Additional Supplementary Files [file 42003_2023_5112_MOESM3_ESM.pdf]

Description of Additional Supplementary Files

File name: Supplementary Data 1

Description: The source data behind the graphs in Figure 1 and Supplementary Figure 1

File name: Supplementary Data 2

Description: The source data behind the graphs in Figure 2 and Supplementary Figure 2

File name: Supplementary Data 3

Description: The source data behind the graphs in Figure 3 and Supplementary Figure 3

File name: Supplementary Data 4

Description: The source data behind the graphs in Figure 4 and Supplementary Figure 4

File name: Supplementary Data 5

Description: The source data behind the graphs in Figure 5 and Supplementary Figure 5
